# UCHL5 is a putative prognostic marker in renal cell carcinoma: a study of UCHL family

**DOI:** 10.1186/s43556-024-00192-0

**Published:** 2024-07-22

**Authors:** Mengdi Zhang, Jingxian Li, Sijia Liu, Fangfang Zhou, Long Zhang

**Affiliations:** 1 Life Sciences Institute, The Second Affiliated Hospital of Zhejiang University School of Medicine, Hangzhou, 310058 PR China; 2 International Biomed-X Research Center, Second Affiliated Hospital of Zhejiang University School of Medicine, Zhejiang University, Hangzhou, 310058 PR China; 3 Key Laboratory of Precision Diagnosis and Treatment for Hepatobiliary and Pancreatic Tumor of Zhejiang Province, Hangzhou, 310058 PR China; 4 The First Affiliated Hospital, the Institutes of Biology and Medical Sciences, Suzhou Medical College, Soochow University, Suzhou, 215123 PR China; 5 The MOE Basic Research and Innovation Center for the Targeted Therapeutics of Solid Tumors, The First Affiliated Hospital, Jiangxi Medical College, Nanchang University, Nanchang, 330031 China; 6 Cancer Center, Zhejiang University, Hangzhou, Zhejiang 310058 PR China

**Keywords:** Ubiquitin C-terminal hydrolase L family, Deubiquitylating enzymes, Immune cell infiltration, Renal cancer, Prognostic marker, Bioinformatics

## Abstract

A macroscopic perspective is indispensable for understanding the intricate relationship between deubiquitinases and tumorigenesis. Proteomics has been proposed as a viable approach for elucidating the complex role of deubiquitylation in cellular progression. Instead of studying the function of a single ubiquitinase, research on a deubiquitinase family with similar catalytic core(s) may provide a new perspective for the pathological understanding of cancer. The Ubiquitin C-terminal hydrolase L (UCHL) family consists of four members: UCHL1, UCHL3, UCHL5, and BRAC1 associated protein-1 (BAP1), and they have been implicated in tumorigenesis and metastasis. Some members are considered hallmarks of intracranial lesions, colon cancer, chromatin remodeling, and histone stability. The present study uncovered an unknown correlation between the UCHL family and renal cancer. We discovered that UCHLs exhibit diverse regulatory effects in renal cancer, establishing connections between the renal cancer and truncated gene mutations, mitochondrial energetic metastasis, immune cell infiltration, and chromosomal stability of UCHLs family. Notably, we found that the increase of UCHL5 expression in renal cancer cells decreases the antigen processing and presentation of RCC tumor-infiltrating B cells. Further research identified that the expression of UCHL5 in RCC tumors is correlated with transport proteins, which led us to find that the abundance of UCHL5 in the blood of late-stage renal cell cancer patients is upregulated from 18 ng/L to 500 ng/L. Therefore, we propose that the abundance of UCHL5 in patients' blood can be a possible indicator of poor prognosis for renal cell cancer.

**Supplementary Information:**

The online version contains supplementary material available at 10.1186/s43556-024-00192-0.

## Highlights

Our study established the unknown relationship between UCHLs and renal cancer tumorigenesis.

UCHL3 and UCHL5 showed immunosuppressive in CD8^+^ T cells and B cells.

The blood abundance of UCHL5 is a putative prognostic marker of RCC.

**Supplementary Information:**

The online version contains supplementary material available at 10.1186/s43556-024-00192-0.

## Introduction

Ubiquitination plays a crucial role in the regulation of all biological processes [1, 2]. The E3 ligase within the ubiquitin system regulates potential substrates by catalyzing the attachment of various ubiquitin(s) to lysine, N-terminal methionine, serine, and threonine residues [3, 4]. To date, more than 700 E3 ligases have been identified, including Ub-conjugating enzymes with E3 ligase activity. Additionally, over 100 deubiquitylating enzymes (DUBs) have been discovered that counteract ubiquitination and dynamically regulate molecular functions and biological processes by hydrolyzing (iso)-peptide bonds between ubiquitins and substrates [5, 6].

DUBs can be classified into nine superfamilies based on their catalytic regions and structural conservation [6]. The Ubiquitin C-terminal hydrolase L (UCHL) family is a typical cysteine protease with a catalytic triad comprising of cysteine, histidine, and aspartate [7, 8]. All members of this family play crucial roles in regulating pathogenesis, particularly tumorigenesis and metastasis [9]. Among them, UCHL1 is abundantly expressed in brain tissues and constitutes up to 5% of the soluble proteins in neuronal cells, despite its low level of mRNA transcription [10]. UCHL1 deficiency through knockout and mutation experiments, demonstrates the crucial role of UCHL1 in maintaining axonal integrity. Biochemical studies have confirmed its acceptance of α- or ε-linked peptides, yet its normal function and physiological substrates remain unknown [11, 12]. Abnormal overexpression in other tissues is also associated with many forms of cancer, including breast and liver cancer [13, 14]. UCHL3 is the closest homologue of UCHL1 in vertebrates, with 53% similarity in humans [15]. However, UCHL3 exhibits distinct tissue expression patterns and functions. It is widely expressed in various tissues, including those of the endocrine system, respiratory system, gastrointestinal tract, liver, and urinary bladder [6]. Its primary function is to maintain chromosomal stability and prevent cancer progression. In addition, recent studies have confirmed that UCHL3 stabilized YES associated protein (YAP) by deubiquitylation, and form a UCHL3/YAP positive feedback loop, thus promoting tumor progression [16]. Structurally, the active site of UCHL3 is pre-arranged in a productive conformation, consistent with the emerging need for DNA repair process [17, 18]. In contrast, the active site of UCHL1 is misaligned, and its enzymatic activity can only be achieved through substrates interaction [7]. The 26S proteasome consists of three subunits: a central 20S proteolytic core particle (CP) and two 19S regulatory particles. The structural preference of the 20S CP prevents erroneous degradation and determines substrate specificity [19]. UCHL5 is a deubiquitinase (DUB) that associates with the 19S subunit of the proteasome to hydrolyze ubiquitin from its substrates [20]. As one of the two DUBs in the ubiquitin–proteasome system along with Ubiquitin specific peptidase 14 (USP14), UCHL5 alternately cleaves ubiquitin chains, specifically targeting the K48 ubiquitin chains, facilitating Ub-recycling [21]. Unlike UCHL1 or UCHL3, which expose the thioester bond between mono-ubiquitin and the substrate for subsequent nucleophilic attack, UCHL5 has the unique ability to process polyubiquitin chains when bound to the proteasome. Its involvement in the degradation of proteins such as β-Catenin, SMAD4, and p65/p50, allows it to regulate cell proliferation and tumorigenesis in various cancers, including gastric cancer, liver cancer, lung cancer, and bladder cancer [22–24]. BRAC1 associated protein-1 (BAP1) is a well-known tumor suppressor. As the largest member of the UCHL family, BAP1 comprises 729 amino acids [25, 26]. Its electronic surface area allows it to interact with various functional proteins, including histones, zinc fingers, and forked transcription factors, such as FoxK1/K2 and YY1 [27, 28]. BAP1 regulates replication fork formation, disrupts S phase arrest, and maintains G1max S phase fidelity under normal cellular conditions [29]. Germline or somatic mutations in BAP1, particularly those in the UCH domain, can result in aggressive malignancies, such as breast cancer and melanoma [30].

Renal cancer refers to 16 types of carcinomas that originate from the kidney tissue, accounting for approximately 4% of all malignancies [31, 32]. It is more prevalent in older males and causes approximately 200,000 deaths per year worldwide [32]. Renal cell carcinoma (RCC) is the most common type of renal cancer, affecting over 90% of patients, and is the most lethal urogenital cancer. RCC can be classified into three subtypes: clear cell carcinoma (KIRC) accounts for 70–90% of cases, papillary cell carcinoma (KIRP) for 10–15%, and kidney chromophobe (KICH) for 3–5%. KIRC and KIRP arise from proximal tubule traits and have a high mortality rate, whereas KICH originates from the distal connecting tubules. Locally advanced RCC may result in distant metastases to the lungs, bones, brain, and liver [33–35]. Given the role of UCHLs family proteins in tumor development and their up-regulation in late RCC, we speculated that UCHLs family proteins may have the potential to be valid indicators for predicting the prognosis of RCC.

Phenotypic plasticity and tumor microenvironment are two categories of cancer hallmarks [36]. These include de-differentiation, differentiation-blocking, and epigenetic reprogramming. Phenotypic plasticity involves genomic aberrations that affect somatic cells and lead to changes in their proliferation and aggressiveness. In contrast, the tumor microenvironment remodels the accessory and immune cells that infiltrate solid tumors or even physical conditions through non-mutational reprogramming to promote tumorigenesis and invasion [37]. The involvement of UCHLs in both processes has been reported individually. For instance, UCHL3 is considered a hallmark of colon cancer in the blood, underscoring its significance in remodeling the tumor microenvironment [38]. However, to date, studies on UCHLs have been limited to individual proteins, lacking integrated research on the entire UCHL family in the context of tumors. In particular, reports on the development of a prognostic diagnosis approach remain insufficient. This study aimed to understand the role of UCHLs in renal cancer from the perspective of the deubiquitome, and investigate the functions of UCHLs through bioinformatic analysis, establish associations between UCHLs and tumorigenesis, and provide potential markers for predicting clinical prognosis.

## Results

### Correlation of mRNA and protein expressions of UCHL family members with renal cancer

**Fig. 1:**
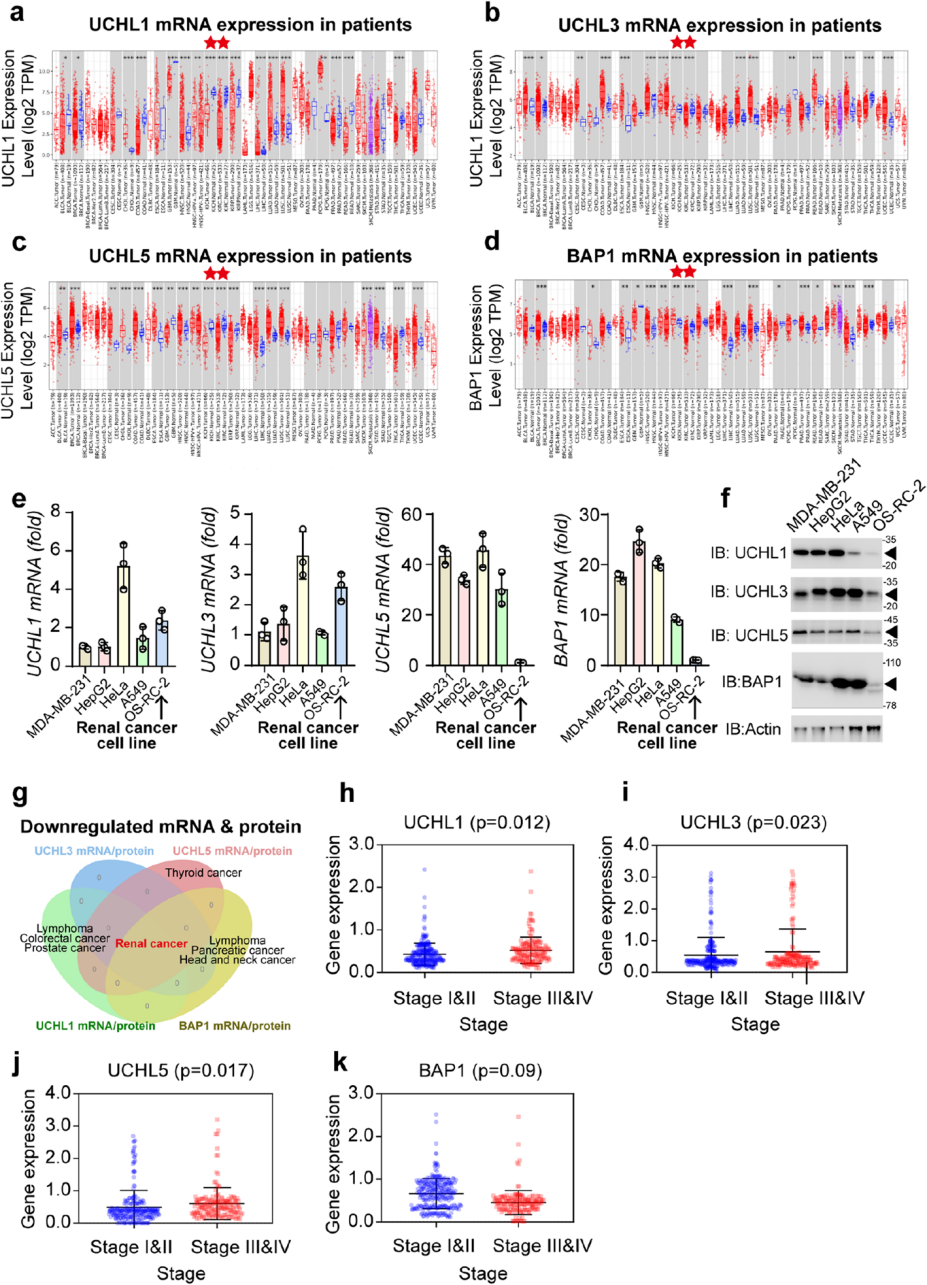
The pan-cancer transcription and expression atlas of the UCHL family.** a–d** Analysis of transcriptional levels of (A) UCHL1, (B) UCHL3, (C) UCHL5, and (D) BAP1 in various tumors from The Cancer Genome Atlas (TCGA). Red stars indicate renal cancer. **e** qPCR analysis of UCHLs mRNA in indicted cell lines. Breast cancer cell line MDA-MB-231, liver cancer cell line HepG2, cervical cancer cell line HeLa, lung cancer cell line A549, and the black arrows indicated renal cancer cell line OS-RC-2, *n* = 3. **f** Immunoblot (IB) of UCHL1, UCHL3, UCHL5, and BAP1 in indicated cell lysates. Actin shows loading amount. **g** Venn graph of different UCHL family members with downregulated mRNA and protein levels across various cancers. UCHL1 is downregulated in lymphoma, colorectal cancer, renal cancer, and prostate cancer; UCHL3 is downregulated in renal cancer; UCHL5 is downregulated in thyroid and renal cancers; and BAP1 is downregulated in lymphoma, pancreatic, and head and neck cancers. Red letters indicate renal cancer. **h–k** The expression of UCHL1, UCHL3, UCHL5, BAP in RCC samples with different total stages. UCHL1 Stage I&II *n* = 214, Stage III&IV *n* = 177; UCHL3 Stage I&II *n* = 210, Stage III&IV *n* = 173; UCHL5 Stage I&II *n* = 203, Stage III&IV *n* = 165; BAP1 Stage I&II *n* = 224, Stage III&IV *n* = 144. Mean ± s.d., statistical analysis was performed using two-tailed Student's *t*-test (**e**)

To investigate the role of UCHLs in pan-cancer, we initiated our study by scrutinizing the correlation between the protein or mRNA levels of UCHLs and various tumor types. As shown in Fig. 1a–d, UCHLs mRNA expression varies across cancers. Results with fewer than 10 samples were excluded because of potential statistical errors. Despite these differences, UCHLs had a consistent decline in KIRC, which was the opposite of the expression pattern in BRAC. Additional research is required to understand the consistency of UCHLs, given their different basic mechanisms. UCHLs also exhibit both up- and down-regulated expression patterns in bladder urothelial carcinoma (BLCA), head and neck squamous cell carcinoma, liver hepatocellular carcinoma (LIHC), and lung adenocarcinoma, involving at least three members of the UCHL family.

To validate the findings of the mRNA investigation, protein expression analysis was performed. This was essential because the correlation between protein and mRNA expression frequently exhibits a nonlinear relationship, especially for degradation-related proteins. For example, UCHL1 protein is notably abundant in brain tissue, constituting almost 3% of soluble proteins in neurons, despite its low mRNA transcript levels. In contrast, the mRNA transcript of UCHL5 is highly abundant in the eyes, but its protein expression is absent [8, 21]. This nonlinear relationship has a greater impact on DUBs because their degradation periods are typically long with unique degradation processes. Traditional scoring results are based on clinical cancer specimens. Protein expression levels were classified as high, medium, or low in comparison to those of normal tissues. A quantitative score was obtained by assigning immunohistochemistry (IHC) results. The down- and upregulation thresholds were set at 25% and 75% of the total patients, respectively. As shown in Fig. S1a–d, protein expression patterns varied among different types of tumors.

UCHL1 expression was negatively correlated with most cancers, except for brain-related gliomas, which may be attributed to its absence in most tissues. UCHL3, the closest homologue of UCHL1, has an expression pattern similar to UCHL1 in cancers. It showed significant negative correlations with most abdominal tumors, except colorectal and testicular cancers. UCHL3 expression is negatively correlated with LIHC, making it a potential prognostic marker [39]. Surprisingly, contrary to previous research on tumor, most cancer types showed a positive correlation with BAP1 expression. BAP1 is a multifunctional transcription factor responsible for the transcription of oncogenes and DNA repair. This finding suggests an unknown mechanism for BAP1 activity. Despite the diversity of UCHLs expression in different tumor types, all of their expressions were negatively associated with renal cancer. qPCR and western blot were used to verify this result. Different types of tumor cell lines, including MDA-MB-231, HepG2, HeLa, A549, and OS-RC-2, were chosen to represent breast cancer, liver cancer, cervical cancer, lung cancer, and renal cancer respectively. Consistently, OS-RC-2 showed similar protein expression patterns of UCHLs as those in patients. Western blot analysis of above cell lines showed slightly different to qPCR result. On protein level, the abundance of UCHLs in OS-RC-2 were lower than others, which further confirmed the reliability of the patient analysis (Fig. 1e, f).

Additionally, there were significant differences in the mRNA and protein expression levels of UCHLs. For example, in liver cancer samples, the mRNA expression of UCHL1 was higher than that in normal samples, while its protein expression was lower. Similarly, in thyroid cancer samples, the mRNA expression level of BAP1 was lower than that in normal samples, but its protein expression was higher. This confirms the inconsistency between the protein and mRNA expression of UCHLs, which aligns with our initial concerns. Despite these differences, all members of the UCHL family exhibited negative mRNA and protein expression in renal cancer, presenting a unique and valuable therapeutic insight (Fig. S1e). It is essential to note that for consistent mRNA and protein expression levels in renal cancer, access to reliable databases will facilitate subsequent analyses. Therefore, we focused on renal cancer in the subsequent UCHL studies and confirmed our findings across different cancer types (Fig. 1g). Meanwhile, by analyzing the expression of UCHLs of RCC samples with different grades and stages, we discovered UCHL1 and UCHL5 are up-regulated in RCC samples Stage III&IV compared with Stage I&II (Fig. 1h-j). The expression of BAP1 is significantly downregulated in Stage III&IV (Fig. 1k). These findings are consistent with overall survival in RCC (Fig. S1f, g). The possibility of UCHLs family proteins as prognostic markers in RCC was suggested.

### BAP1 truncated mutation potentially facilitates RCC development via modulation of the type I interferon signaling (IFN-I) pathway

**Fig. 2:**
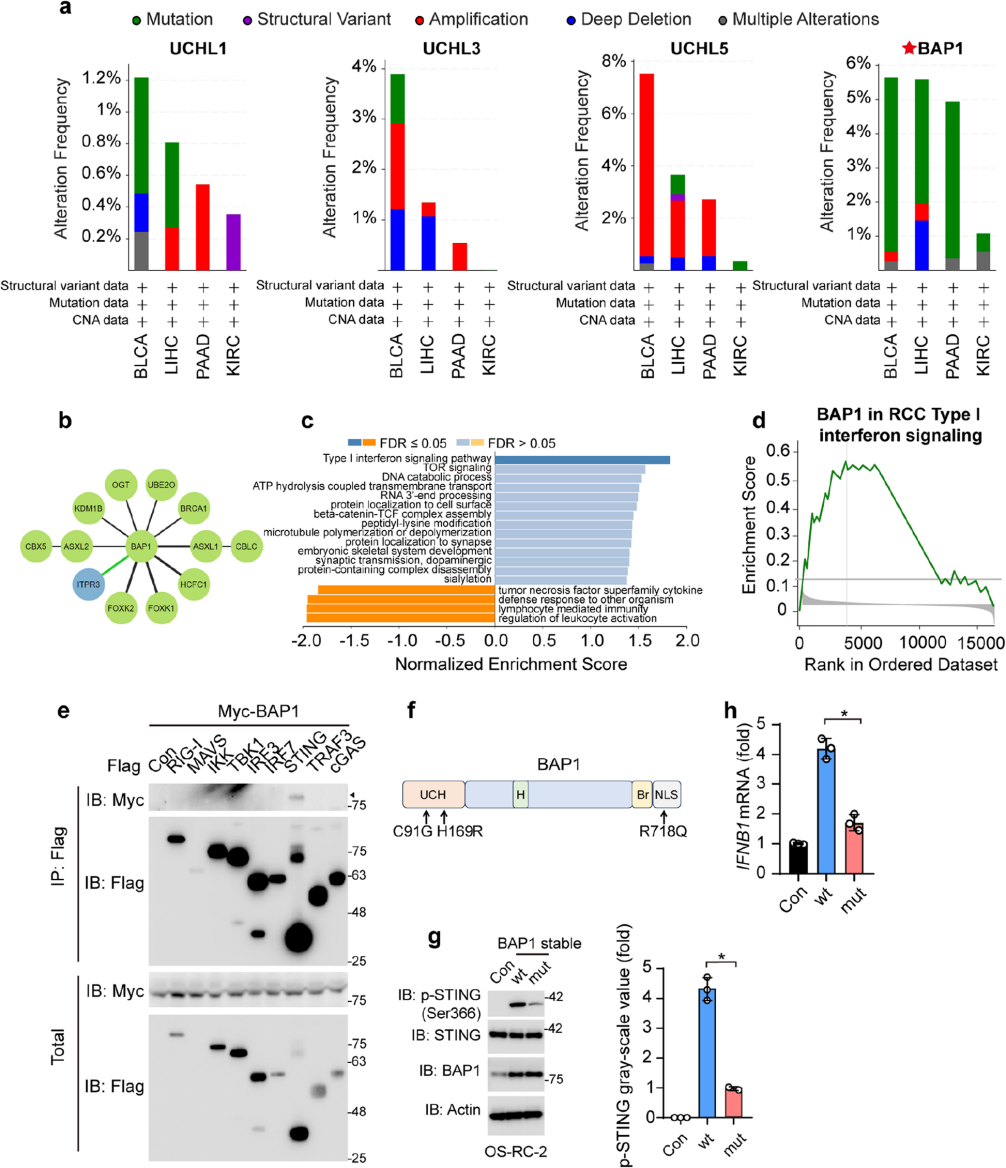
BAP1 truncated mutation potentially facilitates RCC development via modulation of the type I interferon signaling (IFN-I) pathway. **a** The proportion of various UCHL alterations across different subclasses of bladder urothelial carcinoma (BLCA), liver hepatocellular carcinoma (LIHC), pancreatic adenocarcinoma (PAAD), and kidney renal clear cell carcinoma (KIRC). Red stars indicate truncated BAP1 mutations. **b** Protein interactome map of BAP1. **c** Kyoto Encyclopedia of Genes and Genomes (KEGG) pathway analyses of significantly differentially expressed genes correlated with BAP1 expression. The type I interferon signaling (IFN-I) pathway is shown on the top. **d** Gene set enrichment analysis (GSEA) of BAP1 in type I interferon signaling (IFN-I) pathway. **e** Immunoblot (IB) of total and anti-Myc immunoprecipitated (IP) proteins. Control (con.) and flag tagged RIG-I, MAVS, IKK, TBK1, IRF3, IRF7, STING, TRAF3, cGAS were co-transfected with Myc-BAP1 in HEK293T cells. **f** A schematic diagram describing the mutations on BAP1. UCH, ubiquitin C-terminal hydrolase; H, HCF-1-binding motif; Br, BRCA1 binding domain; NLS, nuclear location signal. **g** IB of phosphorylated STING (p-STING Ser366), STING, BAP1, and Actin in OS-RC-2 stably over-expressed BAP1 wt and mutant. Quantified value of p-STING blots grayscale on the right. *n* = 3, *, *p*-value < 0.05. **h** qPCR analysis of *IFNB* mRNA with stably over-expressed BAP1 wt and mutant in OS-RC-2 cell lines. *, *p*-value < 0.05. Mean ± s.d., statistical analysis was performed using two-tailed Student's *t*-test (**h**)

Our primary objective was to establish connections between UCHLs and RCC from a mutational perspective. Given that RCC is a heterogeneous cancer, various factors, including mutations in crucial proteins, contribute to its development. Frequent mutations identified in hereditary clear cell RCC were missense variants and frameshift mutations in Von Hippel–Lindau (VHL) gene [40]. VHL regulates the degradation of hypoxia-inducible factor α and the NF-κB signaling pathway by forming an E3 ligase complex with elongin B and C [41]. However, most cases of RCC are sporadic, implying the accumulation of mutations after birth [42]. In particular, BAP1 has been identified as a potential tumor suppressor in KIRC and type II KIRP. Therefore, the UCHL mutation frequencies in patients with RCCs were compared. The BLCA, LIHC, and PAAD were also examined, as the bladder also belongs to the urogenital system, and the liver and pancreas are organs where RCC tends to metastasize. All UCHLs except BAP1 exhibited a relatively low mutation frequency (less than 1%) in KIRC (Fig. 2a). Truncation mutations in BAP1, disrupting its peptidase region, were the predominant characteristics of kidney cancer.

Additionally, we discovered the underlying biochemical mechanism by which BAP1 suppresses RCC development. Protein–protein interaction analysis and biological process enrichment were performed to investigate the association between BAP and oncoproteins. As shown in Fig. 2b-d, BAP1 was found to be associated with several oncoproteins, including FOXK1/2 and O-GlcNAc transferase, suggesting a potential correlation between dysfunctional BAP1 and oncoproteins. GSEA confirmed that IFN-I signaling is a potential pathway controlling RCC development, as identified through process enrichment. Former research has also proposed that immune activation would impede RCC tumorigenesis [43].To verify this result, we used immunoprecipitation assays to find its binding protein. Myc -BAP is co-transfected into HEK293T cell with Flag tagged proteins in type I interferon signaling pathway. As shown in Fig. 2e, stimulator of interferon genes (STING) was the only interacted protein. This result indicated that BAP1 may regulate IFN-I signaling through STING. To verify the function of BAP1 mutant, we construct truncating mutation mimic by mutating C91G, H169R, and R718Q (Fig. 2f). After lentivirus assembly, OS-RC-2 cell lines were infected for 2 days and screened with puromycin. On the level of STING phosphorylation, the reduced expression in the mutation was detected (Fig. 2g, Fig. S1h). As shown in Fig. 2h, BAP1 wt/mut stably expressed cells also showed opposite results on IFNB1 mRNA level. This means BAP1 wt enhanced the activation of IFN-I signaling pathway, while BAP1 mutant loss this function. In conclusion, our findings suggest that BAP1 suppresses RCC by regulating a series of biological processes, including the STING -involved IFN-I pathway.

### UCHL1 may promote the development of RCC by modulating energy metabolism

**Fig. 3:**
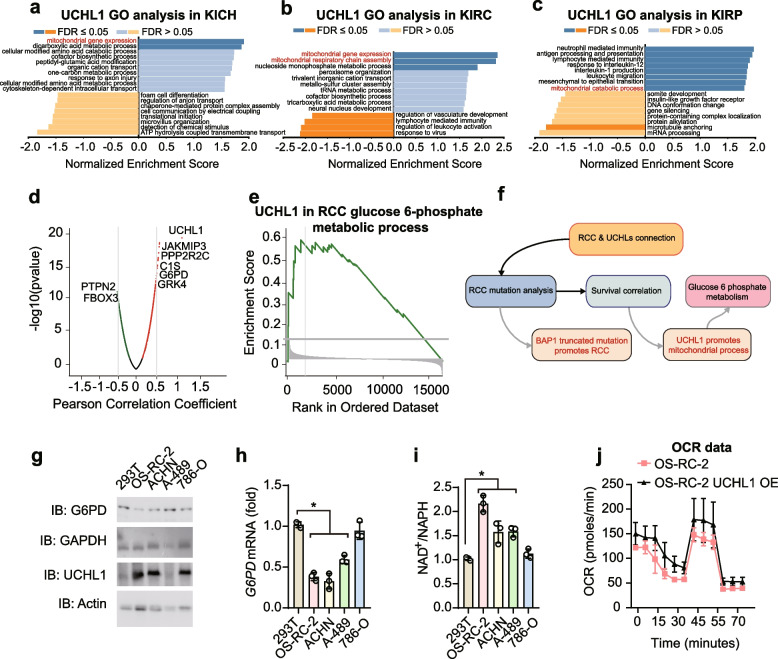
UCHL1 may promote RCC development through energy metabolism. **a–c** Gene Ontology (GO) analysis of significantly differentially expressed genes in correlation with UCHL1 in (E) KICH, (F) KIRC, and (G) KIRP. Mitochondria-related processes are indicated in red. **d** Volcano plot of UCHL1-associated proteins in renal cancers. Metabolism-related proteins are indicated. **e** Gene set enrichment analysis (GSEA) of UCHL1 during glucose-6-phosphate metabolism in RCC. **f** A schematic graph illustrates the connections between RCC and UCHLs. **g** Immunoblot (IB) of G4PD, GAPDH, UCHL1, and Actin in renal cancer cell lines. **h** qPCR analysis of *G6PD* mRNA in indicted cell lines. *, *p*-value < 0.05. **i** The quantified ratio of NAD.^+^/NADH in indicated cells. *, *p*-value < 0.05. **j** Quantified curve of OCR in OS-RC-2 and OS-RC-2 with UCHL1 over-expression. Data are representative of at least three independent experiments (**g**-**j**). Mean ± s.d., statistical analysis was performed using two-tailed Student's *t*-test (**h, i**)

In contrast to BAP1, UCHL1 expression is down-regulated in the early stage and up-regulated in the late stage of RCC. It is also negatively correlated with survival, suggesting that UCHL1 may promote the development of RCC through different mechanisms. Previous studies on the relationship between UCHL1 and the mitochondria have mainly focused on neurodegeneration and mitochondria-mediated apoptosis [44]. It prompted us to perform a Gene Ontology (GO) biological process analysis, which suggested that mitochondrial gene expression and catabolic processes regulate RCC through UCHL1, and any apoptosis-related processes were not identified. However, its involvement in renal cancer has not been reported. Notably, UCHL1 was associated with energetic processes, such as dicarboxylic acid and tricarboxylic acid metabolic processes, as well as antigen processing and presentation (Fig. 3a–c). This underscores the potential involvement of UCHL1 in RCC development. Proteosome correlation enrichment analysis revealed the upregulation of JAKMIP3, PPP2R2C, G6PD, and GRK4, indicating the enhanced activity of these enzymes in metabolism (Fig. 3d). Based on these findings, we hypothesized that UCHL1 exerts its pro-RCC function by enhancing metabolism rather than by directly regulating mitochondrially encoded proteins or apoptosis. Furthermore, GSEA enrichment analysis indicated that glucose-6-phosphate metabolism might be a potential cause of the pro-RCC effect of UCHL1 (Fig. 3e, f). We verified the expression of G6PD and GAPDH in distinct renal cancer cell lines. As showed in Fig. 3g-i, the protein abundance of glucose-6-phosphate dehydrogenase (G6PD) and UCHL1 were negatively correlated, and so as the translational level. To directly verify the function of UCHL1 in aerobic respiration, we tested the OCR data with Seahorse in both of OS-RC-2 and UCHL1 overexpressed OS-RC-2 cells. Consistently, the OCR of OS-RC-2 was lower than which of UCHL1 overexpression in OS-RC-2 (Fig. 3j). This result proved the increasement of UCHL1 in tricarboxylic acid cycle. In summary, the results indicate that the regulation of energy metabolism, particularly glucose-6-phosphate, may have a significant impact on the promotion of UCHL1 in RCC tumorigenesis.

### Tumor-infiltrating T cells are involved in UCHL3-mediated promotion of RCC tumorigenesis

**Fig. 4:**
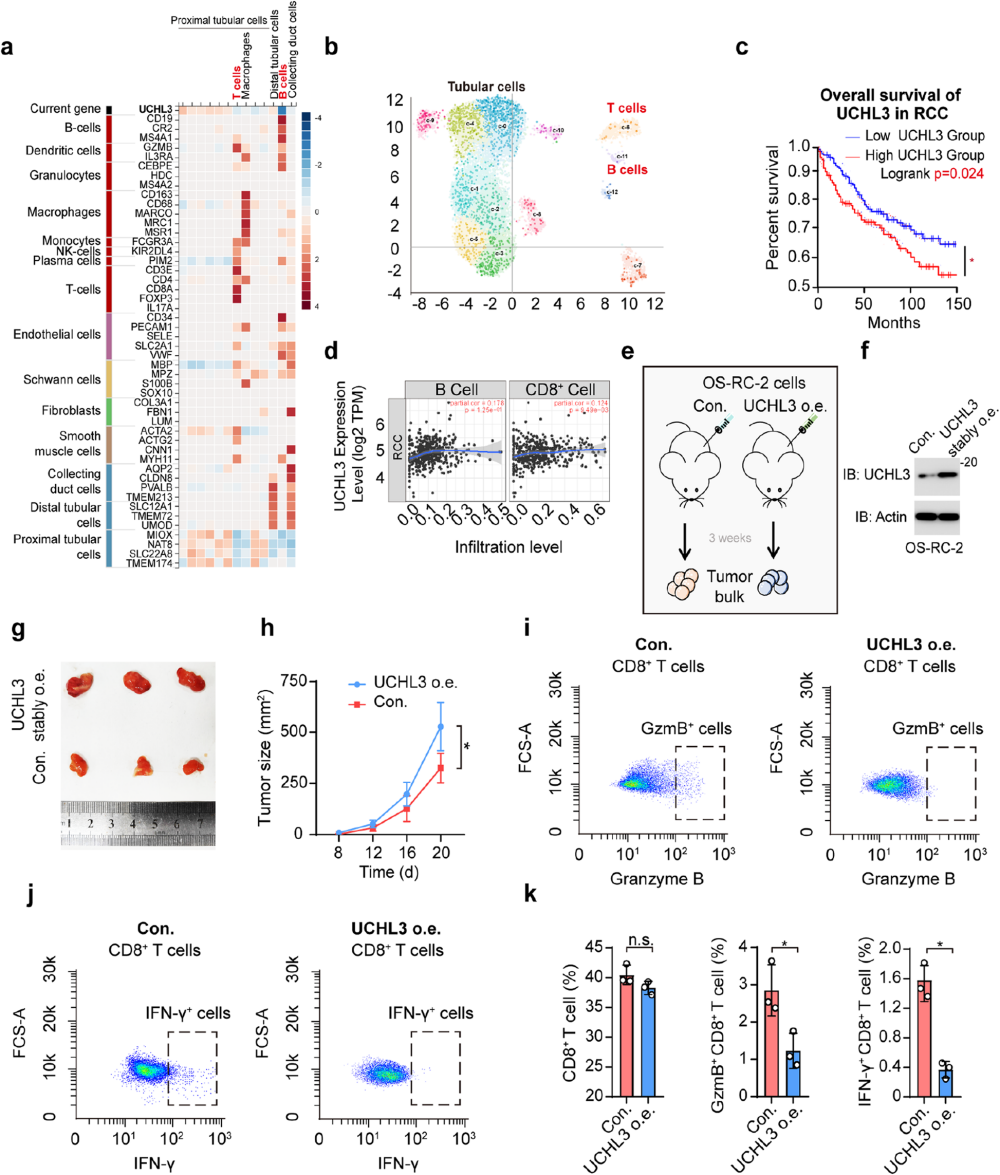
Tumor-infiltrating T cells are involved in UCHL3-mediated promotion of RCC tumorigenesis.** a** Heatmap of the single-cell renal cancer analysis. T and B cells are colored red to show their correlation with UCHL3. The color bar shows the expression levels of the markers in single-cell clusters. **b** Single-cell map of renal cancer tumors. Tubular T and B cells are marked. **c** Survival curves based on UCHL3 expression in patients with RCC. Low UCHL3 group, *n* = 317; High UCHL3 group, *n* = 289. *, *p*-value < 0.05. **d** Correlations between the transcriptional levels of UCHL3 and the infiltration of B cells and CD8^+^ T cells in renal cancer. **e** A schematic diagram of murine subcutaneous model of renal cancer. 10^5^ wt or UCHL3 overexpressed OS-RC-2 cells were subcutaneously injected into mice dorsal area. Tumor sizes were consistently measured, and tumor bulks were separated after 3 weeks. **f** Immunoblot (IB) of UCHL3 and Actin from injected cell in **e**. **g** Macroscopic views of tumour bulks. **h** Quantification of tumour sizes of wt and UCHL3 overexpressed OS-RC-2. *, *p*-value < 0.05. Data are representative of at least three independent experiments. Mean ± s.d., statistical analysis was performed using two-tailed Student's *t*-test. **i** Granzyme B antibody was used to identify CD8^+^ GzmB^+^ T cells (gate: Granzyme B 10^2^–10^3^, FCS-A 5 × 10^2^–5 × 10^3^) from control and UCHL3 overexpressed CD8^+^ T cells separated from tumor. **j** IFN-γ antibody was used to identify CD8^+^ IFN-γ^+^ T cells (gate: IFN-γ 10^2^–10^3^, FCS-A 5 × 10^2^–5 × 10^3^) from control and UCHL3 overexpressed CD8^+^ T cells separated from tumor. **k** Quantified ratio of CD8^+^, GzmB^+^ CD8^+^, IFN-γ^+^ CD8^+^ T cells in flow cytometry. *, *p*-value < 0.05. Data are representative of at least three independent experiments. Mean ± s.d., statistical analysis was performed using two-tailed Student's *t*-test

After identifying the functions of BAP1 and UCHL1, we explored the effects of UCHL3 and UCHL5 in RCC. As shown in Fig. 2a, UCHL3 and UCHL5 regulate RCC tumorigenesis in a non-mutational manner. Therefore, we examined their effects on the regulation of the renal tumor microenvironment (date from cBio Cancer Genomics Portal). We compared the expression of immune markers and UCHL3 in immune and tumor cells using a cell-type marker enrichment analysis [45]. Single-cell analysis of renal tumors demonstrated the high expression of antigen presentation- and cytotoxicity-related genes in infiltrating immune cells, which contrasts with the low level of UCHL3 expression in these cells. In detail, tubular cells constituted the majority of the sample, with a relatively high proportion of T and B cells. UCHL3 expression showed an opposite pattern to that of B cell markers, cluster of differentiation 19 (CD19), complement C3d Receptor 2 (CR2), membrane-spanning 4-domains A1 (MS4A1), and T cell markers, CD3E, CD4, CD8A, and forkhead box P3 (FOXP3) (Fig. 4a, b). Subsequently, we investigated their impact on the microenvironment and distal rehabilitation, particularly on infiltrating immune cells. This finding suggests a negative correlation between UCHL3 expression and RCC-infiltrating T cells.

Since the expression of UCHL3 in total RCC cells decreases overall survival, we further examined the effect of UCHL3 expression in RCC on infiltrating T/B cells (Fig. 4c, d). In infiltrating cells expression assays, we found that UCHL3 expressed in RCC was positively correlated with CD8^+^ T cell, but not B cells (Fig. 4d). Similar results were obtained from GO and GSEA analyses (Fig. S2a, b). UCHL3 showed a negative correlation with leukocyte differentiation, regulation of immune effector processes, regulation of the innate immune response, and T cell activation, all of which are closely associated with infiltrating T cells. To verify this funding, we stably expressed UCHL3 in OS-RC-2 and implanted 10^5^ cells in mice dorsum, and separated tumor bulks after three weeks (Fig. 4e, f). The expression of UCHL3 expanded tumor sizes from about 30 mm^2^ to 500 mm^2^ (Fig. 4g, h). Grinded tumors were subjected to flow cytometry and screened with the expressions of CD45, CD3 and CD8. It is found that the stable expression of UCHL3 had no significant impact on CD8^+^ T cells (Fig. S2c). However, fewer GzmB^+^ CD8^+^ T cells and IFN-γ^+^ CD8^+^ T cells were detected in UCHL3 o.e. tumors compared to control (Fig. 4i-k), which further confirmed that UCHL3 regulates T cell activation, but not T cell proliferation. In conclusion, our findings suggest that UCHL3 promotion of RCC tumorigenesis may be achieved through inactivating CD8^+^ T cells.

### Tumor-infiltrating B cells play a role in UCHL5-mediated promotion of RCC in late stage

**Fig. 5:**
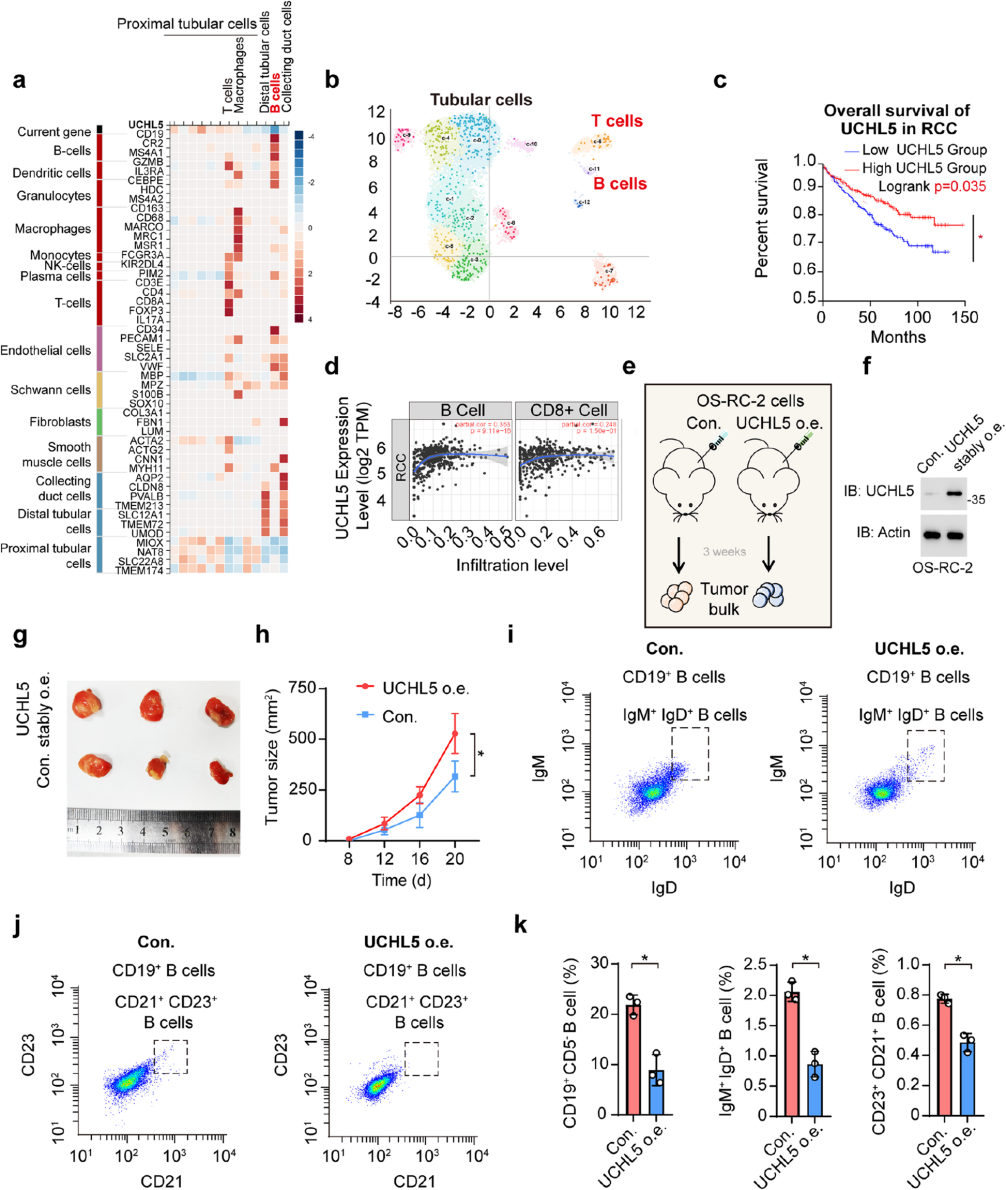
Tumor-infiltrating B cells are involved in UCHL5-mediated promotion of RCC tumorigenesis. **a** Heatmap of single-cell renal cancer analysis B cells are colored red to show their correlation with UCHL5 expression. **b** Single-cell map of renal cancer tumors. Tubular T and B cells are marked. **c** Survival curves based on UCHL5 expression in B cells of patients with RCC. Low UCHL5 group, *n* = 311; High UCHL5 group, *n* = 257. *, *p*-value < 0.05. **d** Correlations between the transcriptional levels of UCHL5 and the infiltration of B cells, CD8^+^ T cells, and CD4^+^ T cells in renal cancer. **e** A schematic diagram of murine subcutaneous model of renal cancer. 10^5^ wt or UCHL5 overexpressed OS-RC-2 cells were subcutaneously injected into mice dorsal area. Tumor sizes were consistently measured, and tumor bulks were separated after 3 weeks. **f** Immunoblot (IB) of UCHL5 and Actin from injected cell in **e**. **g **Macroscopic views of tumour bulks. **h** Quantification of tumour sizes of wt and UCHL5 overexpressed OS-RC-2. *, *p*-value < 0.05. Data are representative of at least three independent experiments. Mean ± s.d., statistical analysis was performed using two-tailed Student's *t*-test. **i**IgM and IgD antibodies were used to identify IgM^+^ IgD^+^ B cells (gate: IgM 1.2 × 10^2^–1.2 × 10^3^, IgD 1.4 × 10^2^–1.2 × 10^3^) from control and UCHL5 overexpressed CD19^+^ B cells separated from tumor. **j** CD21and CD23antibodies were used to identify CD21^+^ CD23^+^ B cells (gate: CD23 1.2 × 10^2^–10^3^, CD21 1.4 × 10^2^–1.2 × 10^3^) from control and UCHL5 overexpressed CD19^+^ B cells separated from tumor. **k** Quantified ratio of CD19^+^ CD5^−^, IgM^+^ IgD^+^, CD23^+^ CD21^+^ B cells in flow cytometry. *, *p*-value < 0.05. Data are representative of at least three independent experiments. Mean ± s.d., statistical analysis was performed using two-tailed Student's *t*-test

A recent article found that PTIR1 binds to the C-terminal of UCHL5 and activates its ubiquitination function, thereby inhibiting immune proteasome activity and limiting the processing and presentation of neoantigens, then preventing T cells from recognizing and attacking cancer [46]. This suggests that UCHL5, like UCHL3, may also promote RCC by regulating immune cell infiltration and activity. In renal cancer IHC samples, the infiltration of immune cells in the RCC tumor was also observed expression of UCHL5, similar to that of UCHL3 (Fig. 5a). Single-cell sequencing also revealed a negative correlation between UCHL5 expression and B cell markers, including CD19, CR2, and MS4A1 **(**Fig. 5a, b). However, unlike UCHL3, UCHL5 expression and T cell markers, such as CD3E, CD4, CD8A, and FOXP3, were positive. This suggests that the mechanism of action of UCHL5 is different from that of UCHL3. Overall survival was significantly downregulated by the expression of UCHL5 in B cells, which indicate that UCHL5 impedes the function of B cells. And the expression of UCHL5 in RCC tumor cells increased B cells infiltration, suggesting an immunosuppressive correlation with B cells but not with T cells (Fig. 5c, d). Proteomics and process enrichment analyses revealed the antigen process and presentation was inhibited (Fig. S3a). UCHL5 expression is negatively correlated with antigen presentation in RCC-infiltrating B cells, as confirmed by GSEA (Fig. S3b). Tumor experiment on mice was executed. Control (Con.) and UCHL5 stably expressed OS-RC-2 were implanted in mice dorsum, and separated tumor bulk after three weeks (Fig. 5e, f). As shown in Fig. 5g, h, UCHL5 expression decreased the tumor size. Meanwhile, the indicators of mature B cells and antigen-representation were both weakened (Fig. 5i-k**, **Fig. S3c). Therefore, we hypothesized that UCHL5 promotes RCC by inhibiting antigen process and presentation in RCC-infiltrating B cells.

### Proposed approach for potential RCC prognosis

As a heterogeneous cancer, hereditary and epigenetic alterations account for less than 3% of RCC cases, with the majority (> 95%) being sporadic [47]. Up to 30% of patients with RCC experience tumor recurrence, so a highly accurate prognostic score is critical for individual monitoring and adjuvant therapy. Among the common prognostic factors, the prognostic model is mainly established according to tumor stage, grade, subtype and clinical characteristics [48]. Laboratory investigations of kidney function are based on the evaluation of complete blood cell counts and urinary analysis of lactate dehydrogenase, calcium, and alkaline phosphatase [49]. However, the accuracy of this conventional prognostic score is limited, and molecular markers can help build a more complete prognostic model, which is the focus of future research.

**Fig. 6:**
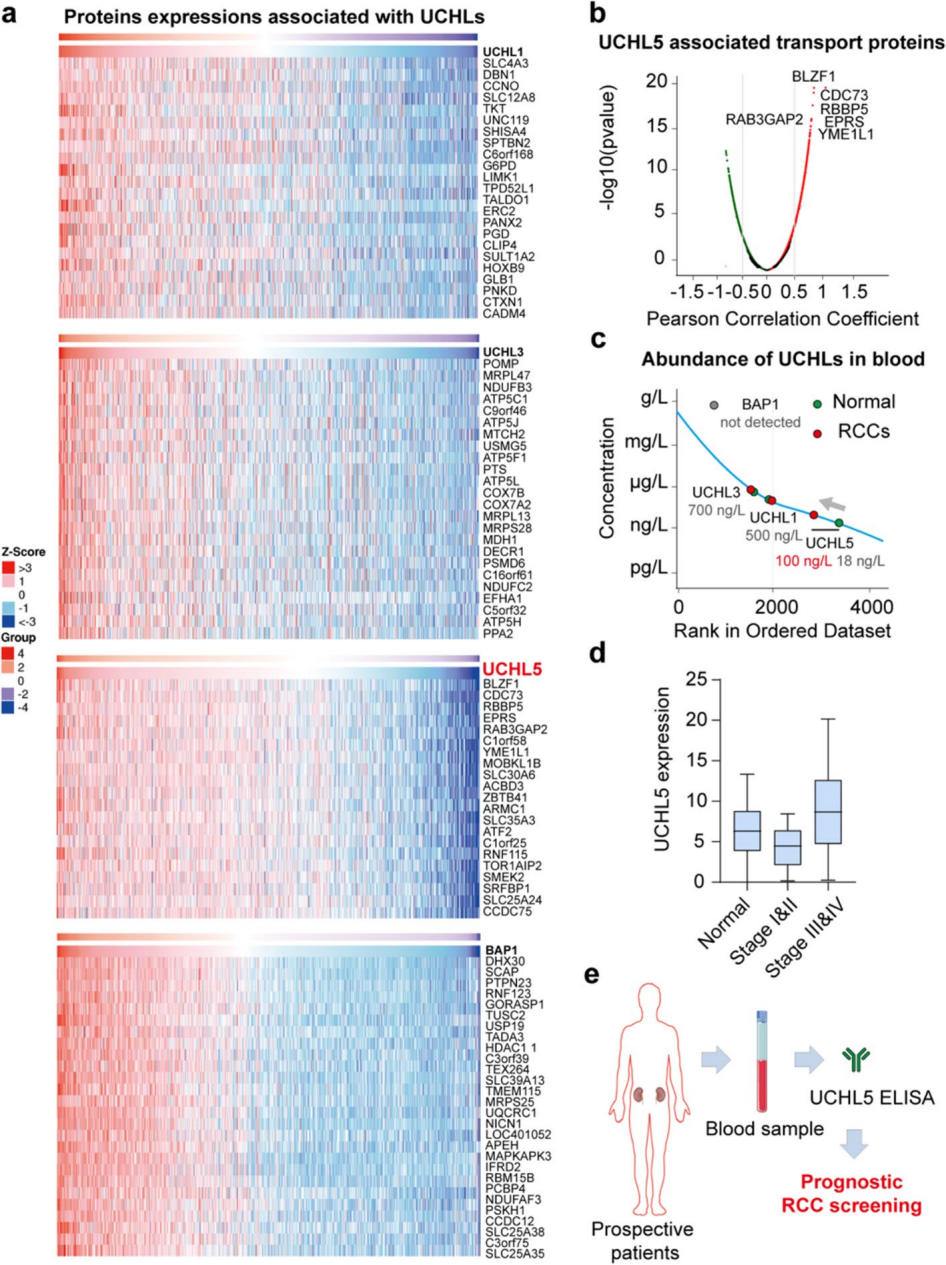
Proposal of UCHL5 as a potential prognosis approach for RCC. **a** Heatmap of UCHL-associated protein mRNA expression levels. UCHL5 is shown in red. **b** Volcano plot of UCHL5-associated transport proteins in RCC. Transport-related proteins are indicated. **c** Abundance of UCHLs in the blood of normal participants and patients with RCC. Green dots indicate the abundance of UCHL1, UCHL3, and UCHL5 in normal blood samples, while red dots indicate the abundance of UCHL5 in RCC blood samples. **d** Boxplot of UCHL5 expression in different RCC stage. *p*-Value: Normal/Stage I&II, 6.2 × 10^–7^; Normal/Stage III&IV, 1.8 × 10^–6^; Stage I&II/ Stage III&IV, 9.4 × 10^–11^. **e** Schematic graph illustrating the potential application of UCHL5 in late stage RCC screening

Through a review of the published literature, BAP1 is frequently mutated in RCC, and its deletion is considered to be an important factor in poor prognosis. The high expression of UCHL1 in cervical squamous cell carcinoma, UCHL3 in ovarian cancer, and UCHL5 in lung adenocarcinoma are also used as markers of poor prognosis [50–53]. Given the scarce reports of UCHL members, excluding BAP1, in renal cancer, and our established connections between them from various perspectives, we aimed to assess the potential of UCHLs for RCC prognosis indicators, especially with a liquid biopsy, which is easier to perform, non-invasive, and faster than the traditional tissue biopsy. Through previous differential gene expression analysis, we found that UCHLs were down-regulated in KIRC, and other UCHLs except BAP1 were also down-regulated in KICH and KIRP. However, when comparing UCHLs expression in RCC samples of different stages, we found that UCHL1 and UCHL5 were up-regulated in late stage of RCC or RCC with higher malignity, suggesting that positive screening of UCHL1/5 may be used as an indicator of poor prognosis of RCC (TCGA database). To determine whether UCHLs can be used as a liquid biopsy marker, we analyzed the potential of UCHLs to be transported into the blood or urine. We analyzed the expression of proteins positively associated with the UCHLs family in RCC samples, as shown in Fig. 6a. It is worth noting that there are many secreted proteins in the samples, such as BLZF1, CDC73, RBBP5, EPRS, RAB3GAP2, YME1L1, etc., which are positively correlated with the expression of UCHL5. (Fig. 6b). This finding demonstrates the prognostic indicator potential of UCHLs, particularly UCHL5, in bodily fluids.

Initially, urine samples were considered for analysis; however, owing to the limited availability of related databases, we opted to analyze the abundance of UCHLs in the plasma. As shown in Fig. 6c, UCHL3 exhibited the highest abundance in the blood, whereas UCHL5 only had a concentration of 18 ng/L in normal samples. However, in blood samples from patients with RCC, the abundance of UCHL5 increased to 100 ng/L, with no significant changes detected in UCHL3 and UCHL1. BAP1 was not detected in either patient or normal samples, which is consistent with findings commonly applied to tissue biopsies. The expression of UCHL5 were upregulated in the late stage of RCC compared to normal samples (Fig. 6d). Meanwhile, considering the damage to the kidneys following RCC, proteins are released into the blood and urine, the presence of UCHL5 in blood at RCC late stage might be a consequence of it. Therefore, we propose that the presence of UCHL5 in the blood may be a potential prognostic marker for RCC (Fig. 6e).

## Discussion

Due to the lack of accuracy of a single prognostic factor, many factors are integrated to form a multivariate prognostic model in clinical practice. Common prognostic factors include anatomic, histological, clinical and molecular factors, of which anatomic and histological prognostic factors are supported by more practical evidence [54]. But the diagnostic capability for common factors in the prognostic examination of RCC is limited [55]. Therefore, molecular markers can be used as supplements to improve the accuracy of prognostic models, action on some small kidney masses, benign lesions such as oncocytoma or angiomyolipoma, leaving patients asymptomatic even in the presence of metastases [56]. Therefore, there is a pressing need to develop a molecular indicator used in liquid biopsy that is convenient for predicting the prognosis of RCC.

The individual functions of UCHLs were identified in this study. The initial objective of this study was to elucidate the genetic relationship between UCHLs and RCC. BAP1 truncated mutations, identified in approximately 15% of patients with RCC, exhibit hindered functionality in maintaining homeostasis of proliferation and replication, including the IFN-I signaling pathway. Since hereditary RCC accounts for less than 5% of all cases, subsequent research has focused on UCHL expression levels. In contrast to the findings of previous studies on apoptosis, the expression of UCHL1 reduced the survival expectations of patients with RCC. This may be due to an increase in mitochondrial catabolic processes, specifically glucose-6-phosphate metabolism. The remodeling of the tumor microenvironment may be one of the factors contributing to the promotion of RCC by UCHL3 and UCHL5. According to the GO analysis, UCHL3 expressed in CD8^+^ T cells could inhibit the activation of RCC-infiltrating T cells, whereas UCHL5 may impede vacuolar transport instead of antigen processing and presentation in B cells infiltrating RCC. UCHL3 and UCHL5 slightly enhanced B and T cell infiltration, which may suggest the poor prognosis in RCC. A significant finding is the increased abundance of UCHL5 in the blood of patients with renal cancer, rising from 18 to 100 ng/L, suggesting its potential as a potential prognostic marker for future therapy.

This study encountered some limitations. The acceptance of only the most significant results of the GSEA and GO analyses may introduce bias and overlook potential factors. As shown in Fig. 3, the processes that were positively correlated with UCHL1 were selected for analysis. However, it is important to not overlook the negative correlation between UCHL1 expression and other biological processes, such as the insulin-like growth factor receptor signaling pathway, as it has been reported to play a role in renal cancer tumorigenesis [57]. Owing to the limited availability of the RCC single-cell database, we were unable to confirm the origin of UCHL5 in the blood. Although kidney damage which may cause complex urogenital symptoms, including proteinuria, may potentially contribute to the elevation of UCHL5 in the blood, this cannot be the sole cause of the dramatic change of UCHL5 in the blood.

It is worth noting that given the universal function of UCHL5 in regulating tumorigenesis [22–24, 53], the upregulation of UCHL5 in the blood may also be related to other cancers. As a constitutive part of proteasome, UCHL5 performs its function by regulating protein degradation. Tumor-related signaling pathways that account on crucial adaptor degradation, including Wnt, NF-κB, and TGF-β are affected by UCHL5, theoretically. Our research has clarified the inhibitory relationship between UCHL5 and RCC-infiltrating B cell antigen presentation but the reason why UCHL5 is upregulated in patient blood remains unknown. Similarly, since the expression of UCHL5 is correlated with various types of tumors, it is a valuable topic to determine whether UCHL5 can be a common marker for cancer screening. Therefore, the relevance, especially the sufficient association, of UCHL5 as a renal cancer marker requires further investigation.

Previous research has shown that targeted inhibition of TA attenuates UCHL5 polyubiquitination catalysis in cancer cells [58]. Meanwhile, the treatment of b-AP15 elevate TRAIL-induced apoptosis in human renal caner Caki cells [59]. The other reports also emphasized the importance of UCHL5 in regulating renal cancer metabolic and cell cycle process [60]. These studies were mainly focused on renal cancer cell and xenografic tumor, reports on patients are quite limited. Related data supported our conclusion indeed, but claiming UCHL5 as a drug target for renal caner seems implausible at this stage. We would like to further investigate TA derivatives in the inhibition of renal cancer to clarify the potential of UCHL5 as a biomarker and drug target in following study. Compared with traditional biopsy, the abnormal blood protein, urine, and blood samples are ideal for large-scale screening. However, owing to limited access to renal cancer and urinary databases, only blood remains a feasible approach for establishing connections between UCHLs and RCC. Additionally, the quantification of UCHL5 was based on mass spectrometry, estimating protein concentrations from peptide accounts. Further studies are required to enhance the credibility of the UCHL5 diagnosis.

To sum up, this study presents a deubiquitinasome-based approach for elucidating RCC tumorigenesis. Single-protein research focusing on underlying mechanisms could overlook the overall relevance of disease and protein functions from a comprehensive perspective. This study presents a novel perspective by focusing on the UCHL family, rather than individual proteins, to determine the tumorigenesis of RCC, and find that the UCHLs family have low expression of mRNA and protein levels in RCC. However, UCHL1 and UCHL5 are up-regulated in KIRC with StageIII&IV. These controversial findings obscure the role of UCHLs in the occurrence or progression of RCC. Through further analysis and experiments, we found that UCHLs share similar catalytic peptides but exhibit contrary functions in RCC. In contrast to BAP1, which inhibits RCC by upregulation of STING activity and activation of interferon, UCHL5 promotes RCC by regulating immune infiltration and antigen presentation of B cells, and its increased level in RCC blood samples suggest its potential as a prognostic marker. Additionally, this study presents a complete workflow for future studies, progressing from inherited gene mutations to protein functions and microenvironment-infiltrating immune cells.

## Materials and methods

### Gene expression profiling interactive analysis

UCHLs expression profiles were analyzed using the Gene Expression Profiling Interactive Analysis tool, which was developed based on The Cancer Genome Atlas (TCGA, https://www.cancer.gov/ccg/research/genome-sequencing/tcga) and Genotype-Tissue Expression (GTEx) databases (Tang et al., 2017, https://www.gtexportal.org/). UCHL expression profiles were compared among different renal cancer types.

### UALCAN

The UALCAN portal (Chandrashekar et al., 2017) was used to analyze the association between the clinical characteristics of patients with renal cancer, to UCHLs expression profiles, and the UCHLs promoter methylation status. Additionally, pan-cancer analysis of UCHL factor expression was also performed using the UALCAN website (http://ualcan.path.uab.edu).

### Kaplan–meier plotter

The relationship between UCHL expression levels and the prognosis of patients with renal cancer was analyzed using the Kaplan–Meier plotter (KM plotter; Gyorffy et al., 2010, https://kmplot.com/analysis/). A total of 2,032 patients were analyzed and divided according to the median expression levels of UCHL transcription factors.

### cBio cancer genomics portal

The alterations in UCHL members in renal cancer subtypes were analyzed using the kidney renal clear cell carcinoma, kidney renal papillary cell carcinoma, Kidney Chromophobe, and Pan-kidney cohort (KICH + KIRC + KIRP) (TCGA, PanCancer Atlas, https://www.cbioportal.org/) datasets, which include information from 512, 283, 65, and 863 samples, respectively. Alteration types include mutations, fusions, amplifications, deep deletions, and multiple alterations.

### LinkedOmics analysis

LinkedOmics (https://linkedomics.org) is a multi-omics database that contains information on 32 cancer types from the TCGA database (Vasaikar et al., 2018). Significantly differentially expressed genes that correlated with UCHL members were analyzed using the TCGA_KICH, TCGA_KIRC, and TCGA_KIRP cancer cohorts (HiSeq RNA platform) in LinkedOmics. Kyoto Encyclopedia of Genes and Genomes (KEGG) pathway analyses were performed using gene set enrichment analysis (GSEA, https://www.genome.jp/kegg/). Genes were classified using Gene Ontology (GO, https://www.geneontology.org/) based on biological processes, cellular components, and molecular functions.

### Tumor Immune Estimation Resource (TIMER) analysis

The TIMER web server (Li B. et al., 2016; Li et al., 2017, http://timer.cistrome.org/) was used to analyze the infiltration of six immune cells type in renal cancer: B cells, CD4^+^ T cells, CD8^+^ T cells, neutrophils, macrophages, and dendritic cells. The gene module was used to evaluate the relationship between target gene expression and immune cell infiltration, whereas the mutation module was used to analyze gene mutations associated with an abundance of immune infiltrates. The SCNA module was used to analyze the correlation between UCHL somatic copy number alterations and tumor infiltration levels.

### Gene set cancer analysis

The Gene Set Cancer Analysis database (Liu C.J. et al., 2018, https://www.gsea-msigdb.org/gsea/index.jsp) was used to analyze the relationship between UCHL factor methylation levels and the infiltration of six immune cell types: B cells, CD8^+^ T cells, CD4^+^ T cells, macrophages, neutrophils, and dendritic cells in renal cancer.

### Lentivirus transduction

Lentiviruses were produced by co-transfecting pLV-STING-GFP, pLV-STING-Flag, pLV-Myc-BAP1, pLV-Flag-UCHL3 or Plv-Flag-UCHL5 plasmids and helper plasmids pCMV-VSVG, pMDLg-RRE (gag/pol), and pRSV-REV into HEK293T or OS-RC-2 cells. Cell supernatants were harvested 48 h after transfection and were used to infect cells or stored at -80 °C. To obtain stable cell lines, cells were infected at low confluence (20%) for 24 h with lentiviral supernatants diluted 1:2 with normal culture medium in the presence of 5 ng/mL polybrene (CAS: 28,728–55-4, Sigma). 48 h after infection, cells were placed under puromycin selection for 1 week and then passaged before use. Puromycin (CAS: 53–79-2, HY-K1057, MCE) was used at 1 μg/mL to maintain selection pressure on stably transfected cells.

### RNA extraction

For cultured cells, total RNA was prepared using the NucleoSpin RNA II kit (Lot: 740,955. 50, BIOKE). A total of 1 μg of RNA was reverse-transcribed using the RevertAid First Strand cDNA Synthesis Kit (Lot: 11,483,188,001, Roche). For mice tissues, excised 4 mm skin biopsies were immediately snap-frozen in liquid nitrogen and stored at − 80 °C until processing. RNA was isolated using the TRIzol/chloroform method and a tissue homogenizer (Lot: 12,183,026, Thermo Fisher Scientific). All isolated RNA had an A260/A280 value ≥ 1.7 and RNA integrity was analyzed on a Fragment analyzer (FA5200, Agilent). Mouse lung pieces were lysed in TRIzol (Lot: 15596018CN, Thermo Fisher Scientific) and RNA was isolated according to the manufacturer’s instructions.

### Quantitative real-time (qPCR)

qPCR was conducted with SYBR Green (Lot: 4,309,155, Applied Biosystems) using a StepOne Plus real-time PCR system (Applied Biosystems). Quantitation of all target gene expression was normalized to the control genes (*Gapdh* for mouse genes or *GAPDH* for human genes). The human primer sequences used were as follows: human *IFNB1* forward, 5′-CCAACAAGTGTCTCCTCCAAAT-3′; human *IFNB1* reverse, 5′-AATCTCCTCAGGGATGTCAAAGT-3′; human *GAPDH* forward, 5′-AGGGCTGCTTTTAACTCTGGT-3′; human *GAPDH* reverse, 5′-CCCCACTTGATTTTGGAGGGA-3′.

### Tumor histology

Dissected tumor bulk were fixed in 10% phosphate-buffered formalin (F5554, Sigma), embedded into paraffin (CAS: 8002–74-2, Sigma), sectioned, stained with hematoxylin and eosin solution (CAS: 517–28-2, Sigma), and examined by light microscopy for histological changes.

### Mouse model

10^5^ wt or UCHL3/5 overexpressed OS-RC-2 cells were subcutaneously injected into mice dorsal area. Tumor sizes were measured every other day, and tumor bulks were separated after 3 weeks for following experiments.

### Immunoblot (IB) analysis

5 × 10^7^ HEK293T cells were transfected with 4 μg Myc-BAP1 and 6 μg indicated proteins from IFN-I signaling pathway, including RIG-I, MAVS, IKK, TBK1, IRF3, IRF7, STING, TRAF3, and cGAS. Cells were harvested in 37 ℃ for 36 h. Cells were lysed with 1 mL lysis buffer (20 mM Tris–HCl, pH 7.4, 2 mM EDTA, 25 mM NaF, and 1% Triton X-100) containing protease inhibitors (I3911, Sigma) for 10 min at 4 ℃. After centrifugation at 12,000 g for 15 min, protein concentrations were measured, and equal amounts of lysates were used for immunoprecipitation. Immunoprecipitation was performed with anti-FLAG M2 beads (A2220, Sigma) for 1 h at 4 ℃, or with various antibodies (identified below) and protein A-Sepharose (Lot: 101,041, GE Healthcare Bio-Sciences AB) for 3 h at 4 ℃. The precipitates were washed three times with washing buffer (50 mM Tris–HCl, pH 8.0, 150 mM NaCl, 1% Nonidet P-40, 0.5% sodium deoxycholate, and 0.1% SDS), and the immunocomplexes were eluted with sample buffer containing 1% SDS for 5 min at 95 ℃. The immunoprecipitated proteins were separated thereafter by SDS-PAGE. Immunoblot analysis was performed with specific antibodies (identified below) and secondary anti-mouse or anti-rabbit antibodies conjugated to horseradish peroxidase (HRP) (identified below). Rabbit mAb to Actin (Abclonal AC026, 1:50,000 for IB), rabbit polyclonal anti-Myc (A-14) (Santa Cruz Biotechnology, sc-789; 1:2000 for IB), mouse monoclonal anti-Myc (9E10) (Santa Cruz Biotechnology, sc-40; 1:2000 for IB), protein A-HRP (Sigma-Aldrich, GENA9120; 1:10,000 for IB) and HRP-conjugated secondary antibodies (Cell Signaling, 7076 (anti-mouse IgG) or 7074 (anti-rabbit IgG); 1:10,000 for IB). Rabbit mono-clonal antibody to total UCHL1 (ABclonal A19101, 1:1000 for IB); Rabbit poly-clonal antibody to total UCHL3 (ABclonal A8156, 1:1500 for IB); Rabbit mono-clonal antibody to total UCHL5 (ABclonal A23792, 1:1000 for IB); Rabbit mono-clonal antibody to total BAP1 (ABclonal A24830, 1:1500 for IB), HRP-conjugated secondary antibodies 7074 (anti-rabbit IgG); 1:10,000 for IB; STING/TMEM173 Rabbit mAb (ABclonal A21051, 1:1500 for IB), Phospho-STING/TMEM173-Ser366 Rabbit mAb (ABclonal AP1369, 1:1500 for IB).

### Flow cytometry

For all in vitro tumor bulk cell assays, cells were washed with PBS 2% FBS twice, stained with surface staining markers (diluted 1:200) at room temperature for 20 min, washed twice and resuspended in PBS 2% FBS with DRAQ7 or APE (diluted 1:1,000) before analysis on a Beckton Dickinson FACSymphony X-50 flow cytometer. The strategy for identifying B cell and T cell subsets using surface markers and FACS is as follows. SSC-A and FCS-A were used to verify the viability with 3 μL of 50 μg/mL DAPI staining. CD45 was used to categorize immune cells (gate: CD45 5 × 10^2^–10^4^, FCS-A 0–20 k). CD3 and CD11b antibodies were used to separate T cells (gate: CD11b 0–10^2^, CD3 5 × 10^2^–5 × 10^3^). CD8^+^ CD4^−^ T cells (gate: CD8 100–500, CD4 10–10^2^) were identified as CD8^+^ T cell. Granzyme B^+^ T cells (gate: Granzyme B 10^2^–10^3^, FCS-A 5 × 10^2^–5 × 10^3^). IFN-γ^+^ T cells (gate: IFN-γ 10^2^–10^3^, FCS-A 5 × 10^2^–5 × 10^3^). CD5 and CD19 antibodies were used to categorize CD19^+^ CD5^−^ B cells (gate: CD5 0–10^3^, CD19 1.2 × 10^3^–10^4^). IgM^+^ IgD^+^ B cells (gate: IgM 1.2 × 10^2^–1.2 × 10^3^, IgD 1.4 × 10^2^–1.2 × 10^3^). CD21^+^ CD23^+^ B cells (gate: CD23 1.2 × 10^2^–10^3^, CD21 1.4 × 10^2^–1.2 × 10^3^). Antibody information is as follows: CD45 (ab317446, ABclonal), CD3 (ab135372, ABclonal), CD11b (ab8878, ABclonal), CD8 alpha (ab217344, ABclonal), CD4 (ab207755, ABclonal), CD5 (ab300120, ABclonal), CD19 (ab245235, ABclonal), IgM (ab190369, ABclonal), IgD (ab235126, ABclonal), CD21 (ab227662, ABclonal), CD23 (ab315289, ABclonal), GzmB (ab317458, ABclonal), IFN-γ (ab171081, ABclonal).

### Cells

HEK293T, HeLa, OS-RC-2, A489, 786-O, HepG2, A549, MDA-MB-231 cell lines obtained from the American Type Culture Collection were cultured at 37 °C under 5% CO_2_ in DMEM/F-12 (Lot: 11,965,126, ThermoFisher), MEM (Lot: A1451801, ThermoFisher), or RPMI-1640 (Lot: 11,875,168, ThermoFisher) medium supplemented with 10% fetal bovine serum (Lot:A5669401, Gibco), 100 U/mL penicillin and 100 μg/mL streptomycin (Lot:15,140,122, ThermoFisher).

### Statistics

The exact value of n, representing the number of patients in the experiments, is indicated in the figure legends. Statistical differences with a *P* value of 0.05 or less were considered significant. Specific *p* value and experimental repeats were detailed in related figure legends.

## Supplementary Information

Supplementary Material 1.

## Data Availability

The datasets analysed during the current study are available in the TCGA repository, https://www.cancer.gov/tcga
