# Correction: UCHL5 is a putative prognostic marker in renal cell carcinoma: a study of UCHL family

**DOI:** 10.1186/s43556-026-00449-w

**Published:** 2026-04-27

**Authors:** Mengdi Zhang, Jingxian Li, Sijia Liu, Fangfang Zhou, Long Zhang

**Affiliations:** 1 Life Sciences Institute, The Second Affiliated Hospital of Zhejiang University School of Medicine, Hangzhou, 310058 PR China; 2 International Biomed‑X Research Center, Second Affiliated Hospital of Zhejiang University School of Medicine, Zhejiang University, Hangzhou, 310058 PR China; 3 Key Laboratory of Precision Diagnosis and Treatment for Hepatobiliary and Pancreatic Tumor of Zhejiang Province, Hangzhou, 310058 PR China; 4 The First Affiliated Hospital, the Institutes of Biology and Medical Sciences, Suzhou Medical College, Soochow University, Suzhou, 215123 PR China; 5 The MOE Basic Research and Innovation Center for the Targeted Therapeutics of Solid Tumors, The First Affiliated Hospital, Jiangxi Medical College, Nanchang University, Nanchang, 330031 China; 6 Cancer Center, Zhejiang University, Hangzhou, Zhejiang 310058 PR China

**Correction: Mol Biomed 5, 28 (2024)**

**https://doi.org/10.1186/s43556-024-00192-0**

Following the publication of the original article [1], it is reported that the GAPDH in Fig. 3g had been incorrectly presented, which resulted from an inadvertent lapse in file management and figure preparation. Specifically, the exposure image of UCHL3 in Fig. 1f was mistakenly used for the GAPDH panel in Fig. 3g. The GAPDH band in Fig. 3g has been replaced with the experimental data attached to this correct version. This change does not alter the core data and analytical logic of the original experiment, and does not affect the established research results and conclusions of the original article.

Fig. 3 has been corrected from:

**Fig. 3:**
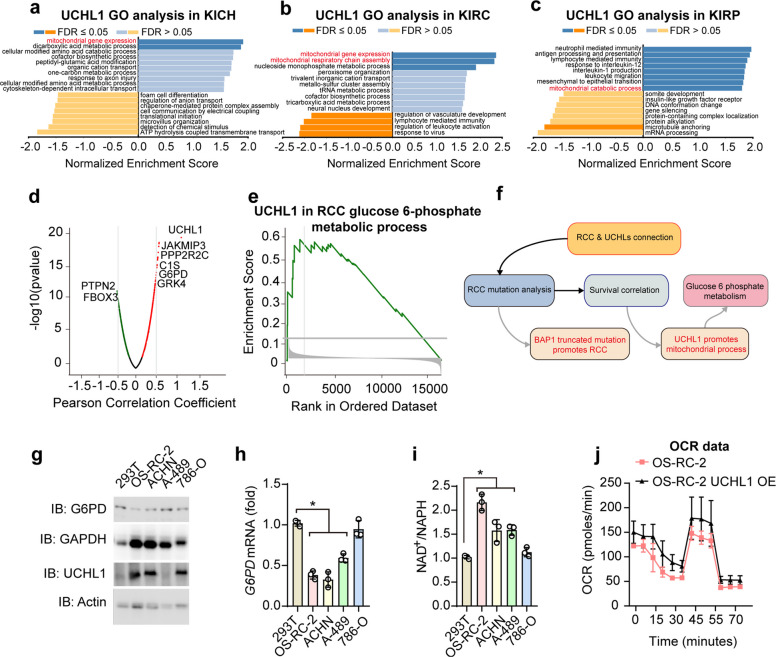
UCHL1 may promote RCC development through energy metabolism. **a–c** Gene Ontology (GO) analysis of significantly differentially expressed genes in correlation with UCHL1 in (E) KICH, (F) KIRC, and (G) KIRP. Mitochondria-related processes are indicated in red. **d** Volcano plot of UCHL1-associated proteins in renal cancers. Metabolism-related proteins are indicated. **e** Gene set enrichment analysis (GSEA) of UCHL1 during glucose-6-phosphate metabolism in RCC. **f** A schematic graph illustrates the connections between RCC and UCHLs. **g** Immunoblot (IB) of G4PD, GAPDH, UCHL1, and Actin in renal cancer cell lines. **h** qPCR analysis of *G6PD* mRNA in indicted cell lines. *, *p*-value < 0.05. **i** The quantified ratio of NAD.^+^/NADH in indicated cells. *, *p*-value < 0.05. **j** Quantified curve of OCR in OS-RC-2 and OS-RC-2 with UCHL1 over-expression. Data are representative of at least three independent experiments (**g**-**j**). Mean ± s.d., statistical analysis was performed using two-tailed Student's *t*-test (**h, i**)

To:

**Fig. 3:**
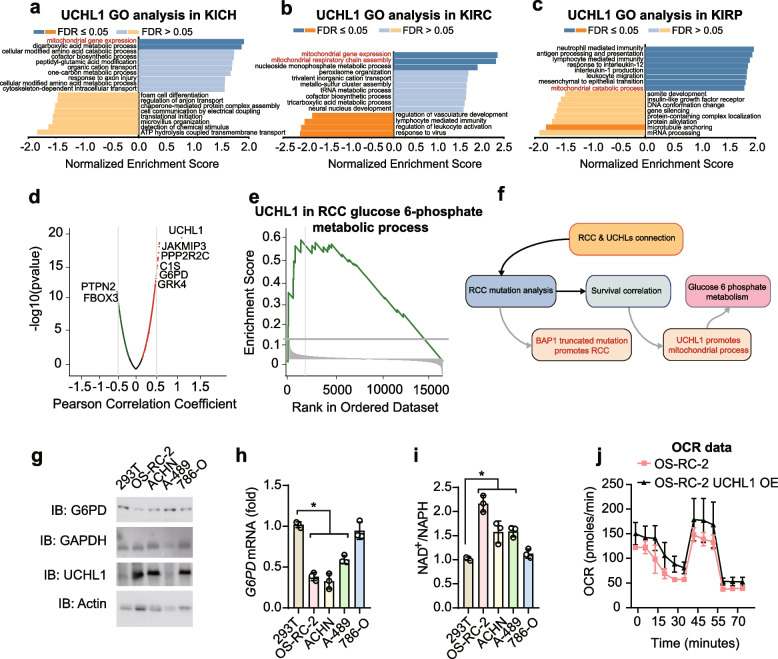
UCHL1 may promote RCC development through energy metabolism. **a–c** Gene Ontology (GO) analysis of significantly differentially expressed genes in correlation with UCHL1 in (E) KICH, (F) KIRC, and (G) KIRP. Mitochondria-related processes are indicated in red. **d** Volcano plot of UCHL1-associated proteins in renal cancers. Metabolism-related proteins are indicated. **e** Gene set enrichment analysis (GSEA) of UCHL1 during glucose-6-phosphate metabolism in RCC. **f** A schematic graph illustrates the connections between RCC and UCHLs. **g** Immunoblot (IB) of G4PD, GAPDH, UCHL1, and Actin in renal cancer cell lines. **h** qPCR analysis of *G6PD* mRNA in indicted cell lines. *, *p*-value < 0.05. **i** The quantified ratio of NAD.^+^/NADH in indicated cells. *, *p*-value < 0.05. **j** Quantified curve of OCR in OS-RC-2 and OS-RC-2 with UCHL1 over-expression. Data are representative of at least three independent experiments (**g**-**j**). Mean ± s.d., statistical analysis was performed using two-tailed Student's *t*-test (**h, i**)

The original article [1] has been updated.
