# Supplementary material for: UCHL5 is a putative prognostic marker in renal cell carcinoma: a study of UCHL family
Source: Mol Biomed. 2024 Jul 22;5:28. doi: 10.1186/s43556-024-00192-0 (PMC11265068; doi:10.1186/s43556-024-00192-0)
Supplement: Supplementary file 1 — Supplementary Material 1. [file 43556_2024_192_MOESM1_ESM.pdf]

UCHL5 is a putative prognostic marker in renal cell carcinoma: A study of UCHL family

Mengdi Zhang[1], Jingxian Li[1], Sijia Liu[2, 3], Fangfang Zhou[4], Long Zhang [1, 5, 6 *]

[1] Life Sciences Institute, The Second Affiliated Hospital of Zhejiang University School of Medicine, The MOE Key Laboratory of Biosystems Homeostasis & Protection and Zhejiang Provincial Key Laboratory for Cancer Molecular Cell Biology, Zhejiang University, Hangzhou 310058, PR China.

[2] International Biomed-X Research Center, Second Affiliated Hospital of Zhejiang University School of Medicine, Zhejiang University, Hangzhou, China.

[3] Key Laboratory of Precision Diagnosis and Treatment for Hepatobiliary and Pancreatic Tumor of Zhejiang Province, Hangzhou, China.

[4] Institutes of Biology and Medical Science, Soochow University, Suzhou 215123, PR China.

[5] The MOE Basic Research and Innovation Center for the Targeted Therapeutics of Solid Tumors, The First Affiliated Hospital, Jiangxi Medical College, Nanchang University, Nanchang 330031, China.

[6] Cancer Center, Zhejiang University, Hangzhou, Zhejiang 310058, PR China.

*** Correspondence:**

Corresponding Author: L.Z (L_Zhang@zju.edu.cn)

This PDF file includes:

1. Supplementary Figures

2. Supplementary Figure Legends

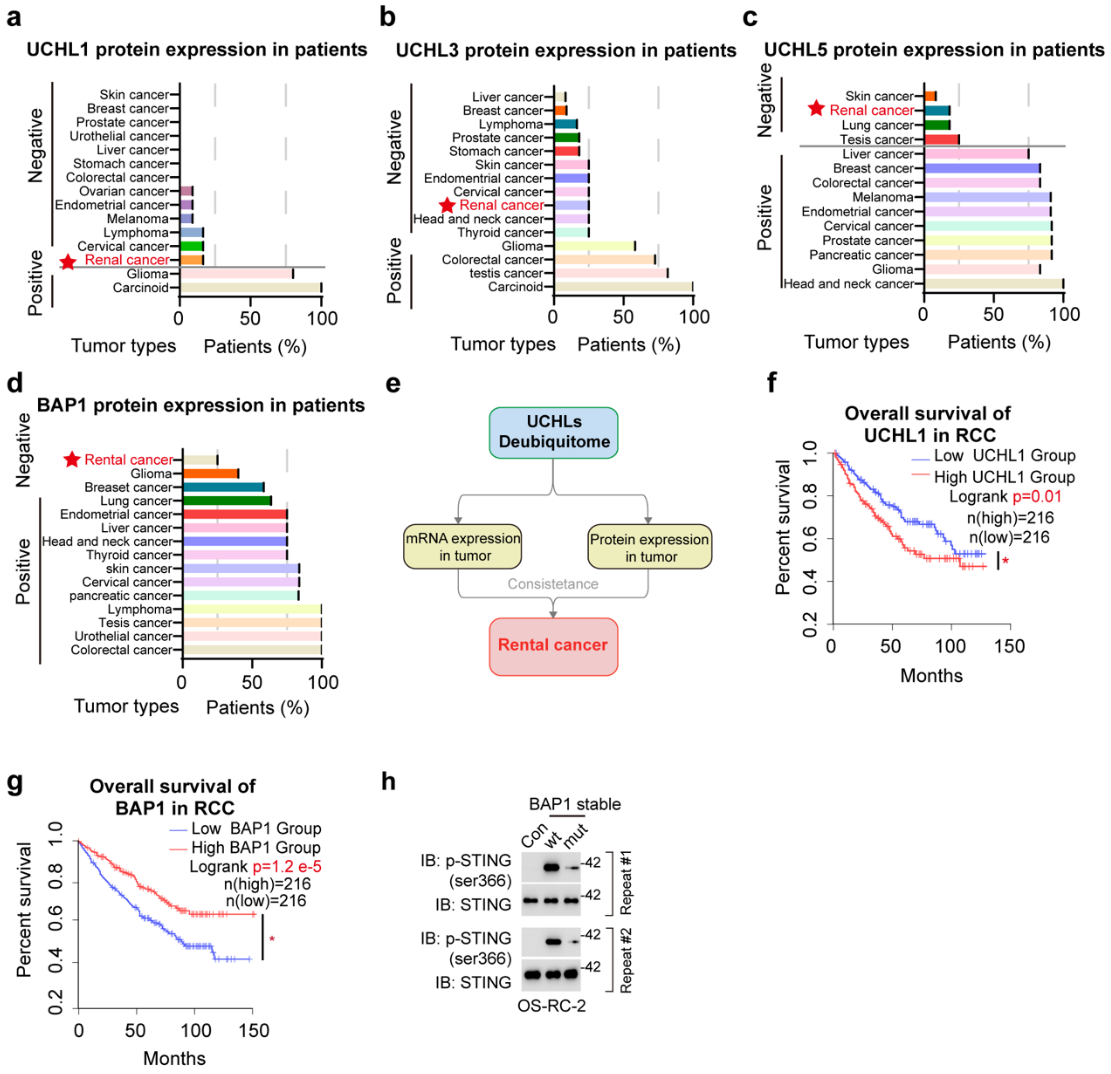

Supplementary figure 1

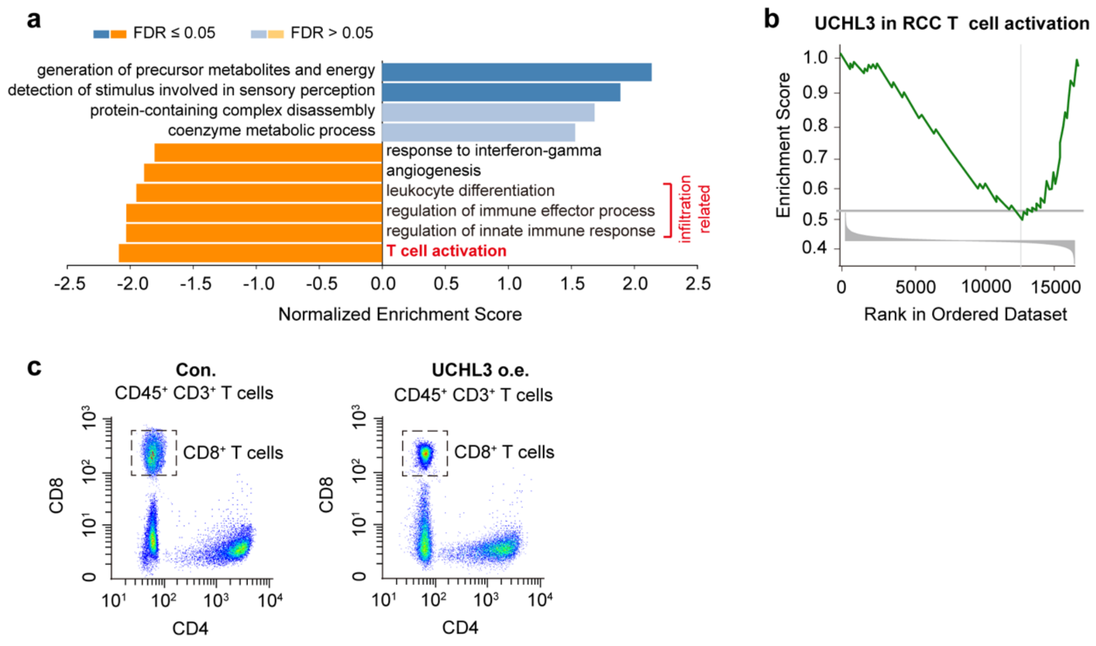

Supplementary figure 2

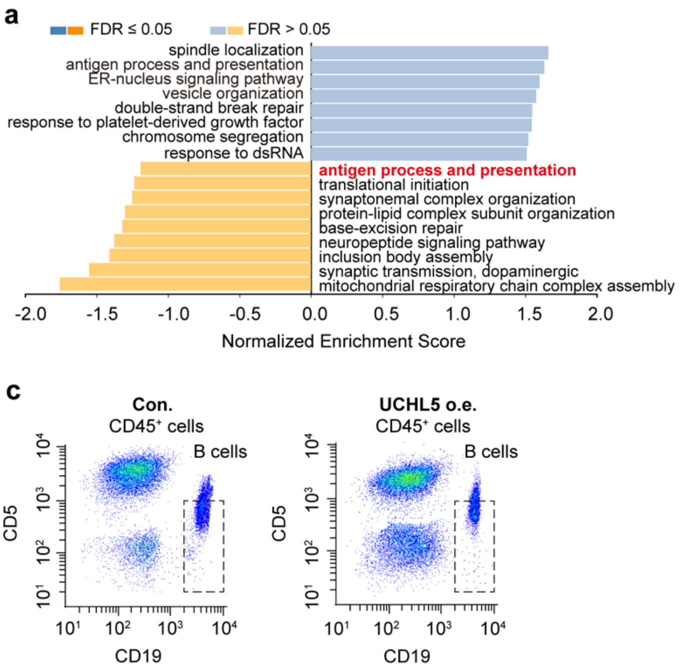

Supplementary figure 3

Supplementary Fig. 1 | The pan-cancer transcription and expression atlas of the UCHL family.

a–d: Comparison of protein expression of (a) UCHL1, (b) UCHL3, (c) UCHL5, and (d) BAP1 across different types of tumors from the TCGA database. Red stars indicate renal cancer. Positive and negative values refer to the relationship between UCHL proteins expression and the percentages of different tumor types.

e: Schematic graph of UCHL deubiquitome screening in renal cancer.

f, g: Survival curves of (f) UCHL1, (g) BAP1 in patients with RCC. Low UCHL1 group, n= 153; High UCHL1 group, n= 148 (f); Low BAP1 group, n= 286; High BAP1 group, n= 244 (g). *, p-value <0.05.

h: IB repeat of phosphorylated STING (p-STING Ser366) and STING in OS-RC-2 stably over-expressed BAP1 wt and mutant as in Fig. 2g.

Supplementary Fig. 2 | Tumor-infiltrating immune cells are involved in UCHL3 mediated promotion of RCC tumorigenesis.

a: Gene Ocotology (GO) analysis of significantly differentially expressed genes correlated with UCHL3 expression. T cell activation is shown in red. Leukocyte differentiation, the regulation of immune effector processes, and innate immune responses are markedly related to infiltration.

b: Gene set enrichment analysis (GSEA) of UCHL3 in RCC with T cell activation.

c: CD8 and CD4 antibodies were used to identify $CD8^+$ $CD4^-$ T cells (gate: CD8 100-500, CD4 10-10^2) from control and UCHL3 overexpressed $CD45^+$ $CD3^+$ T cells separated from tumor.

Supplementary Fig. 3 | Tumor-infiltrating immune cells are involved in UCHL5 mediated promotion of RCC tumorigenesis.

a: Gene Ocotology (GO) analysis of significantly differentially expressed genes correlated with UCHL5 expression. Antigen process and presentation is shown in red.

b: Gene set enrichment analysis (GSEA) of UCHL5 in RCC-infiltrating B cells with antigen process and presentation.

c: CD5 and CD19 antibodies were used to identify $CD19^+$ $CD5^-$ B cells (gate: CD5 0-10^3, CD19 1.2×10^3-10^4) from control and UCHL5 overexpressed $CD45^+$ B cells separated from tumor.
